# Identification of ABA-Mediated Genetic and Metabolic Responses to Soil Flooding in Tomato (*Solanum lycopersicum* L. Mill)

**DOI:** 10.3389/fpls.2021.613059

**Published:** 2021-03-05

**Authors:** Carlos De Ollas, Miguel González-Guzmán, Zara Pitarch, José Tomás Matus, Héctor Candela, José Luis Rambla, Antonio Granell, Aurelio Gómez-Cadenas, Vicent Arbona

**Affiliations:** ^1^Departament de Ciències Agràries i del Medi Natural, Universitat Jaume I, Castelló de la Plana, Spain; ^2^Institute for Integrative Systems Biology, Universitat de València – Consejo Superior de Investigaciones Científicas, Paterna, Spain; ^3^Instituto de Bioingeniería, Universidad Miguel Hernández, Elche, Spain; ^4^Instituto de Biología Molecular y Celular de Plantas, Universitat Politècnica de València – Consejo Superior de Investigaciones Científicas, València, Spain

**Keywords:** abscisic acid, hypoxia, metabolism, signaling, soil flooding, tomato

## Abstract

Soil flooding is a compound abiotic stress that alters soil properties and limits atmospheric gas diffusion (O_2_ and CO_2_) to the roots. The involvement of abscisic acid (ABA) in the regulation of soil flooding-specific genetic and metabolic responses has been scarcely studied despite its key importance as regulator in other abiotic stress conditions. To attain this objective, wild type and ABA-deficient tomatoes were subjected to short-term (24 h) soil waterlogging. After this period, gas exchange parameters were reduced in the wild type but not in ABA-deficient plants that always had higher *E* and *g*_*s*_. Transcript and metabolite alterations were more intense in waterlogged tissues, with genotype-specific variations. Waterlogging reduced the ABA levels in the roots while inducing PYR/PYL/RCAR ABA receptors and ABA-dependent transcription factor transcripts, of which induction was less pronounced in the ABA-deficient genotype. Ethylene/O_2_-dependent genetic responses (ERFVIIs, plant anoxia survival responses, and genes involved in the N-degron pathway) were induced in hypoxic tissues independently of the genotype. Interestingly, genes encoding a nitrate reductase and a phytoglobin involved in NO biosynthesis and scavenging and ERFVII stability were induced in waterlogged tissues, but to a lower extent in ABA-deficient tomato. At the metabolic level, flooding-induced accumulation of Ala was enhanced in ABA-deficient lines following a differential accumulation of Glu and Asp in both hypoxic and aerated tissues, supporting their involvement as sources of oxalacetate to feed the tricarboxylic acid cycle in waterlogged tissues and constituting a potential advantage upon long periods of soil waterlogging. The promoter analysis of upregulated genes indicated that the production of oxalacetate from Asp *via* Asp oxidase, energy processes such as acetyl-CoA, ATP, and starch biosynthesis, and the lignification process were likely subjected to ABA regulation. Taken together, these data indicate that ABA depletion in waterlogged tissues acts as a positive signal, inducing several specific genetic and metabolic responses to soil flooding.

## Introduction

Extreme adverse environmental conditions affect worldwide crop production, among which floods are the most common natural hazards in the world (Voesenek and Bailey-Serres, 2015). It is estimated that nearly 16% of the world’s arable land is currently affected by soil waterlogging, and these numbers are likely to increase associated to a higher incidence of heavy rains due to climate change (Vidoz et al., 2010). Indeed: according to Voesenek and Bailey-Serres (2015), the most important agricultural areas of the world will experience an increase in the incidence of soil waterlogging and subsequent reduction in crop production.

Soil waterlogging is a compound stress that limits the access of plants to vital atmospheric gases such as O_2_ and CO_2_ by replacing air-filled soil spaces with water and restricting gas diffusion to plant tissues (Müller et al., 2019). Moreover, under these conditions, soil undergoes electrochemical changes that favor the presence of reduced forms of elements such as Mn^2+^ or Fe^2+^ and different forms of sulfide (H_2_S, HS^–^, and S^2–^) (Voesenek and Bailey-Serres, 2015). Hence, aerated shoots exhibit a reduction in photosynthetic activity and transpiration (*E*) associated to a reduction in root hydraulic conductivity (*L*_*pr*_) that limits water flow to the aerial part, resulting in leaf turgor loss (Rodríguez-Gamir et al., 2011). On the other hand, waterlogged tissues experience a rapid shift from aerobic to anaerobic metabolism, with the induction of genes encoding proteins involved in this process such as pyruvate decarboxylase (PDC), alcohol dehydrogenase (ADH), and sucrose synthase aimed to maintain energy supply to drive growth and ensure survival. These responses are regulated by ethylene (ET) through the induction of group VII of ethylene response factors (ERFVIIs) homologous to rice SUB1A which confers submergence tolerance to this crop (Hinz et al., 2010). In *Arabidopsis thaliana*, this family comprises five members: RAP2.2 (related to AP2.2), RAP2.3, RAP2.12, hypoxia-responsive element 1 (HRE1), and HRE2 (Hess et al., 2011). The activity of these transcription factors is regulated by balancing synthesis (induced by ET) and targeted proteasomal degradation. This is achieved *via* recognition and covalent modification of destabilizing residues in their N-terminus (or N-degron) carried out in successive steps by plant cysteine oxidases (PCOs) and Arg–tRNA protein transferases (ATE) in the presence of O_2_. Finally, those residues are tagged for proteasomal degradation by the action of specific E3 ligases such as PROTEOLYSIS6 (PRT6) (Gibbs et al., 2011; Vicente et al., 2017). In the absence of O_2_, PCOs within the N-degron pathway are not operative, acting as true oxygen sensors, which subsequently stabilize ERFVIIs that induce the specific hypoxia-specific responses mentioned above.

Besides ET, nitric oxide (NO) has been recently characterized as an important regulator of responses in hypoxic tissues along with phytoglobins (formerly class-1 non-symbiotic hemoglobins) (Gupta et al., 2011; Vicente et al., 2017). NO is a gaseous signal molecule that exerts its regulatory activity through post-translational covalent modification of proteins (namely, *S*-nitrosylation, Tyr-nitration, and metal nitrosylation). Particularly, Cys *S*-nitrosylation has been shown to control a number of key regulatory proteins during plant abiotic stress responses associated to abscisic acid (ABA) signaling such as the NADPH oxidase RESPIRATORY BURST OXIDASE HOMOLOG D (RBOHD), SUCROSE NON-FERMENTING1-RELATED PROTEIN KINASE 2.6/OPEN STOMATA 1 (SnRK2.6/OST1), and several members of the PYRABACTIN RESISTANCE1/PYR1-LIKE/REGULATORY COMPONENTS OF ABA RECEPTORS (PYR/PYL/RCAR) ABA receptor family (Castillo et al., 2015; Yeung et al., 2018). In addition, it has been recently shown that different elements of the ABA signaling pathway might be subjected to transcriptional regulation by ERFVIIs in the presence of NO in *Arabidopsis* (León et al., 2020).

Abscisic acid has long been acknowledged as a key regulator of plant responses to abiotic stress conditions such as drought, salinity, or elevated temperatures as its accumulation controls multiple gene response networks, leading to tolerance and adaption to varying abiotic stress conditions (Gomez-Cadenas et al., 2015; Zandalinas et al., 2016). However, a drastic depletion in the endogenous levels of ABA has been reported in submerged tissues of different plant species, including the model plant *Arabidopsis thaliana* (Hsu et al., 2011) and important crops such as citrus (Arbona and Gómez-Cadenas, 2008; Argamasilla et al., 2013) and tomato (Else et al., 1995; Sharp et al., 2000). ABA decline is mediated by the ET-dependent induction of ABA 8′-hydroxylase and the conversion of ABA to phaseic acid (PA) in submerged tissues (Benschop et al., 2007; Saika et al., 2007; Shimamura et al., 2014; Arbona et al., 2017). In most studied plant models, ABA has been assigned a negative regulator role of flooding-induced responses such as secondary aerenchyma formation in soybean (Shimamura et al., 2014) or the emergence of adventitious root in tomato (Sharp et al., 2000). However, Müller et al. (2019) have recently demonstrated that underwater stem elongation in watercress *Nasturtium officinale* is primarily driven by ABA decline. Moreover, in *Arabidopsis* (Liu et al., 2005) and citrus (Arbona et al., 2017), hypoxic conditions have been linked to the overexpression of genes involved in ABA responses, particularly specific ABA receptors and ABA-dependent transcription factors, suggesting that this plant hormone could have a role in the regulation of responses of plants to flooding-induced hypoxia through its targeted depletion, potentially constituting a flooding-specific response. Nevertheless, the specific genetic and metabolic processes regulated by ABA in waterlogged tissues are still poorly known.

Therefore, the main objective in this work was to characterize the physiological function of ABA depletion occurring in hypoxic tissues as a specific response to soil flooding, leading to the regulation of plant adaptive genetic and metabolic responses. Owing to its economic importance, the amount of work existing on responses to soil flooding, the availability of ABA-deficient lines (*flacca*, *notabilis*, or *sitiens*, *etc*.), and the technical feasibility of studying local *vs.* systemic responses to soil flooding conditions, tomato (*Solanum lycopersicum* L. Mill) has been chosen as a model. To attain this objective, physiological, metabolic, and transcriptional data were analyzed and integrated from the perspective of functional ABA signaling in hypoxic tissues.

## Materials and Methods

### Plant Material, Growth, and Stress Conditions

The tomato (*Solanum lycopersicum* L. Mill) cultivars Lukullus (Luk) and Ailsa Craig (AC), along with their isogenic ABA-deficient lines *notabilis* (*not*), affected in 9-*cis*-epoxycarotenoid dioxygenase (NCED) gene that converts 9-*cis*-neoxanthin to xanthoxin (Thompson et al., 2000), and *flacca* (*flc*), lacking the molybdenum cofactor required for ABA aldehyde oxidase 3 (AAO3) activity (Cornish and Zeevaart, 1988), were used. Tomato seeds obtained from the Tomato Genetics Resource Centre (TGRC, University of California, Davis, United States) were surface-sterilized with a sodium hypochlorite solution (2% v/v), rinsed, and subsequently germinated in porexpan trays on a mixture of peat moss and vermiculite (80:20) in the greenhouse under natural photoperiod and temperature (15:9 h and 24/18°C, day/night temperature) and ambient relative humidity (45–65%). At 2 weeks after germination, the individual plants were transplanted to 2-L plastic pots containing a mixture of peat moss/perlite/vermiculite (80:10:10) and watered regularly, alternating water and Hoagland nutrient solution (Hoagland and Arnon, 1950). The seedlings were maintained in these conditions for 2 months before the beginning of the experiments. In general, the seedlings of ABA-deficient genotypes showed a slower development and were smaller in size (Supplementary Figure S1A).

Soil flooding was imposed by submerging the entire rootzone in tap water contained in plastic-sealed dark pots (Supplementary Figure S1B). During the stress period, O_2_ concentration in flooding water was monitored with a portable dissolved O_2_ meter (Hanna instruments S.L, Guipuzkoa, Spain). The immersed pots were immobilized by placing a heavy weight on the substrate to avoid floating and re-oxygenation (Supplementary Figure S1B). Gas exchange parameters (stomatal conductance, *g*_*s*_, and transpiration, *E*) and leaf water potential (LWP, expressed in MPa) were measured on intact and excised leaves on third position (from the shoot apex) from five individual plants. For details on the measurement of physiological parameters please, refer to Arbona et al. (2017) and De Ollas et al. (2018).

Fully expanded leaves in an intermediate position in the stem and young lateral roots from each individual plant replicate were harvested, rinsed with water, blotted dry, and frozen in liquid N_2_. The frozen plant material from each biological replicate was ground to fine powder and stored at −80°C for RNAseq, hormonal, and metabolite analyses.

### RNA Isolation, RNAseq, and Identification of Differentially Expressed Genes

Total RNA was extracted from frozen plant tissue with Qiagen RNeasy minikit (Qiagen, Valencia, CA, United States) following the manufacturer’s instructions. Total RNA quality was assessed on final extracts with Nanodrop^TM^ (Fisher Scientific, United States) and Bioanalyzer^TM^ (Agilent Technologies, United States). The libraries for RNASeq were prepared from 3 μg of total RNA using the TruSeq Stranded mRNA LT Sample Prep Kit (Illumina Inc., San Diego, CA, United States) and pair-end sequenced using the NovaSeq 6000 platform, producing 151 nt-long reads. Bioinformatics analysis was carried out using the Artificial Intelligence RNASeq (AIR) platform^1^ as in Ferreira et al. (2019). The quality of the raw reads was checked with FastQC and sequence trimming to remove low-quality bases, and sequencing adaptors were performed with the tool BBDuk. High-quality reads (Phred-like score = 25 and minimum read length = 25 nt) were mapped against the *Solanum lycopersicum* SL3.0 reference genome with STAR software. Read summarization was performed with featureCounts using high-quality mapping reads. Genes with very low expression were filtered using HTSFilter, and differentially expressed genes (DEGs) were identified with edgeR after normalization by the trimmed mean of *M*-values method [genes meeting a corrected *p*-value and false discovery rate (FDR) lower than 0.05]. Gene Ontology Enrichment Analysis was performed following a hypergeometric test on the proportion of GO categories between the DEGs and the whole genome (enriched GO categories meeting a FDR value lower than 0.05) (Supplementary Files S1, S2). Identification of gene expression trend clusters was achieved by subjecting root DEGs to hierarchical cluster analysis (HCA) using fragments per kilobase of transcript per million mapped reads (FPKM) values and setting Euclidean distance as the distance metrics and “complete” as the clustering method. Three independent biological replicates were used for RNAseq analyses. Genes belonging to clusters showing different trends between Luk and *not* were selected for subsequent promoter transcription factor recognition motif enrichment analysis.

### Promoter Transcription Factor Recognition Motif Enrichment Analysis

Promoter sequences from genes included in trend clusters defined after HCA were retrieved in FASTA format from biomart^2^ from the *Solanum lycopersicum* SL3.0 dataset including only protein-coding sequences and selecting 1,000-bp 5′ upstream of the transcription start site. Using a custom BioPERL script, the promoter sequences were screened for conserved hexamer motifs, and their abundance in the selected gene clusters were compared with that of all protein-coding genes in the tomato genome. The specific enrichment was expressed as [(*n* of a specific hexamer in the promoters of genes within a given cluster list)/(*n* of different hexamers in the promoters of genes within a given cluster list)/(*n* times a specific hexamer appears in the genome)/(*n* times different hexamers appear in the genome)]. The statistical significance of enrichment values was assessed after a hypergeometric test as in Ma et al. (2013) and corrected following the Benjamini–Hochberg FDR procedure. In selected gene clusters, all hexamer motifs showing an enrichment fraction equal or above 1.1 and a *p*-value meeting the significance criteria (*p*-values equal or lower than the calculated FDR) were searched against the PLACE plant *cis*-acting regulatory DNA elements database^3^ for known *cis*-regulatory motifs. Only positively matching motifs are presented.

### Plant Hormone Profiling

Analysis of plant hormones (ABA, PA, JA, and JA-Ile) was attained with LC/ESI-MS/MS (Waters Acquity SDS UPLC coupled to a TQ-D, Micromass Ltd.) on aqueous plant extracts (c.a. 100 mg fresh weight for each replicate) as in De Ollas et al. (2018). Hormone quantitation was performed with Masslynx v. 4.1 software (Micromass Ltd., United Kingdom) after external calibration with standard samples containing known amounts of each plant hormone. Four independent biological replicates were used for plant hormone measurements.

### Polar Metabolite Profiling

Polar metabolites were determined by GC/EI-TOF-MS (Agilent 6890N gas chromatograph coupled to a LECO Pegasus 4D TOF mass spectrometer) profiling of derivatized extracts (c.a. 50 mg fresh frozen plant material per biological replicate; four independent biological replicates were used). Identification of metabolites was achieved by comparison of mass spectra and retention time with known standards co-injected in the same conditions. The peak area of each identified compound was normalized to internal standard area (ribitol, added on extraction) and sample weight with ChromaTOF software as in Zanor et al. (2009).

### Semipolar Metabolite Profiling

Analysis of specialized metabolites with semipolar chemical characteristics was achieved with RPLC/ESI-QTOF-MS (Waters Acquity SDS UPLC coupled to a QTOF Premier, Micromass Ltd.). Mass chromatographic feature extraction, alignment, and ion annotation were performed with xcms and CAMERA software as in Zandalinas et al. (2017). Compound annotation was achieved by comparison of mass spectra and retention time with those of authentic standards when available or by matching spectral information with that available in databases (metlin, Massbank or HMDB, see Supplementary File S3). Four independent biological replicates were used for plant semipolar metabolite analyses.

### Statistical Analyses

The physiological parameters were analyzed by two-way ANOVA using genotype (G) and treatment (T) as factors and considering also their interaction (G × T). Given the differences existing between genotypes in terms of hormonal levels and to focus on the particular effect of flooding, for representation purposes, data were normalized to control values set as 100%. Nevertheless, two-way ANOVA was performed on absolute hormonal values. When the factor was significant, a least significant difference *post hoc* test was performed to compare sample means.

Differences in metabolite profiles were assessed first by partial least squares discriminant analysis (Simca-P+, Umetrics Ltd., Umea) and a subsequent two-tailed Student’s *t*-test between control and treated samples to investigate specific differences. Unless otherwise stated, statistical analyses were carried out with Statgraphics Centurion XVI 16.0 (Statpoint Technologies, Inc., Warrenton, VA, United States).

## Results

### Characterization of the Physiological Response of Wild Type and ABA-Deficient Tomato Plants to Soil Flooding

**FIGURE 1:**
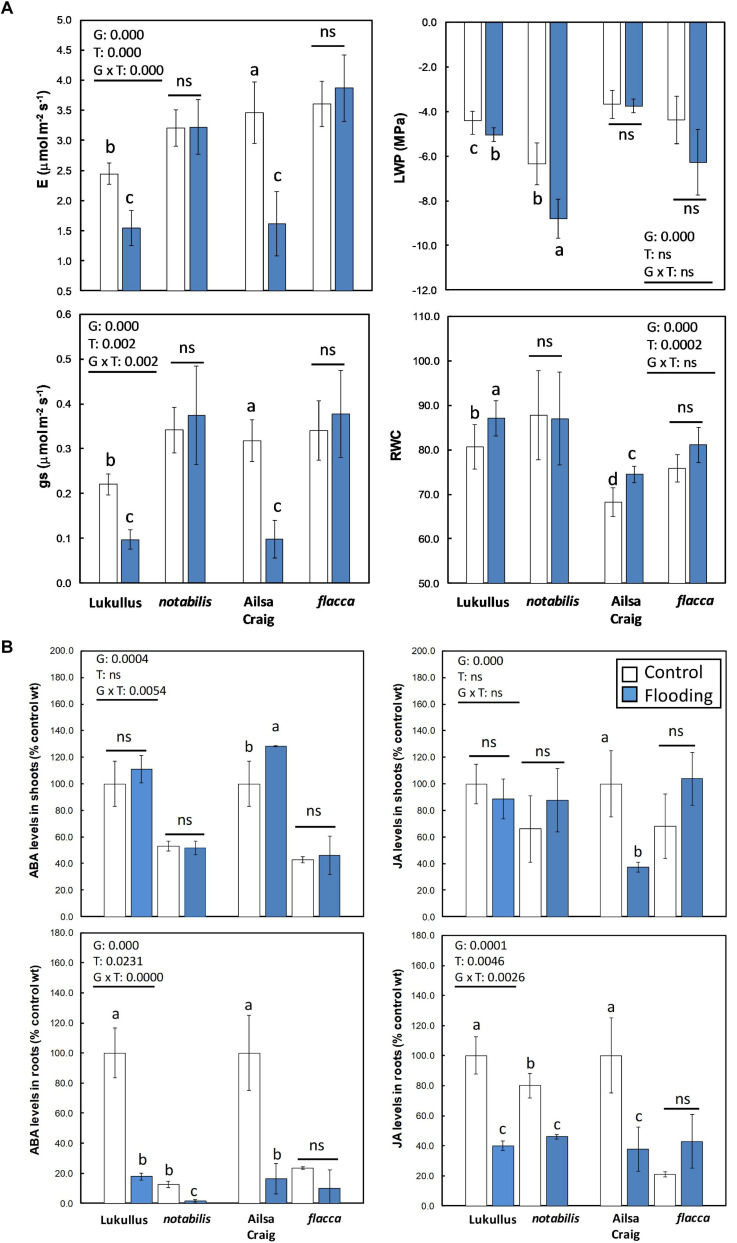
Physiological characterization of wild type (Lukullus and Ailsa Craig) and their respective ABA-deficient isogenic lines *notabilis* and *flacca* tomato plants subjected to soil flooding: gas exchange parameters along with leaf water potential and leaf relative water content **(A)** and hormonal profiling in shoots and roots (*N* = 4) **(B)**. Bars in white and blue are control and flooded groups, respectively. Data are mean values of (*N* = 5) independent measurements. Statistical differences were assessed following a two-way ANOVA using genotype and treatment as factors. Different letters denote significant differences between means at *p* ≤ 0.05 after a least significant difference *post hoc* analysis. ns, non-significant comparisons.

After 24 h of continuous soil flooding, the dissolved O_2_ levels ranged between 4.1 and 4.3 mg l^–1^. In a first series of experiments, two *S. lycopersicum* cultivars, Luk and AC, along with their isogenic ABA-deficient lines, *not* and *flc*, were subjected to short-term soil flooding, and their physiological traits and hormonal profiles were compared (Figure 1 and Supplementary Figure S1). Irrespective of their specific genetic background, both wild type genotypes exhibited a reduction in gas exchange parameters, *E* and *g*_*s*_, and a slight increase in relative water content (RWC) when subjected to soil flooding, whereas the ABA-deficient genotypes did not show any significant alteration in these parameters (Figure 1A). Interestingly, only the Luk/*not* combination showed a significant reduction in LWP in response to soil flooding. It is worthwhile noting that, in general, the ABA-deficient mutants always showed lower LWP than their wild types, even under non-stressful conditions (Figure 1A). Regarding leaf RWC, both ABA-deficient mutants did not exhibit any differences with respect to the controls, whereas Luk and AC showed an increase in this parameter (Figure 1A). The levels of ABA and JA in shoots did not show any significant alteration in response to soil flooding in the Luk/*not* pair, whereas in the AC/*flc* tandem, only the wild type showed a significant accumulation of ABA and depletion of JA (Figure 1B). Nevertheless, in roots, a clearer trend was observed. In both genotype combinations, soil flooding induced an overreduction of ABA, whereas only *not* plants showed a significant reduction of JA levels. In the Luk/*not* pair, ABA depletion in roots was accompanied by a significant accumulation of PA, and the reduction in JA levels in *not* plants followed a parallel reduction in JA-Ile content (Supplementary Figure S2). Finally, an analysis of typical hypoxia-associated metabolites Ala, Asp, and sucrose was attained (Supplementary Figure S3). In general, the metabolite levels in AC/*flc* were higher than in Luk/*not*, but the trends were almost identical: an accumulation of Ala in waterlogged tissues, along with a reduction in Asp, which also showed higher levels in ABA-deficient mutants than in wild types. Only sucrose showed a variable behavior somehow associated to the specific genetic impairment leading to ABA deficiency. As a result of this set of preliminary experiments, the combination Luk/*not* was chosen for subsequent experiments, owing to the less pleiotropic effect of the *not* mutation on physiological parameters and similarity to previous hormonal results obtained in citrus (Arbona and Gómez-Cadenas, 2008; Arbona et al., 2017).

### Characterization of the Genetic and Metabolic Response of Tomato to Soil Flooding

**FIGURE 2:**
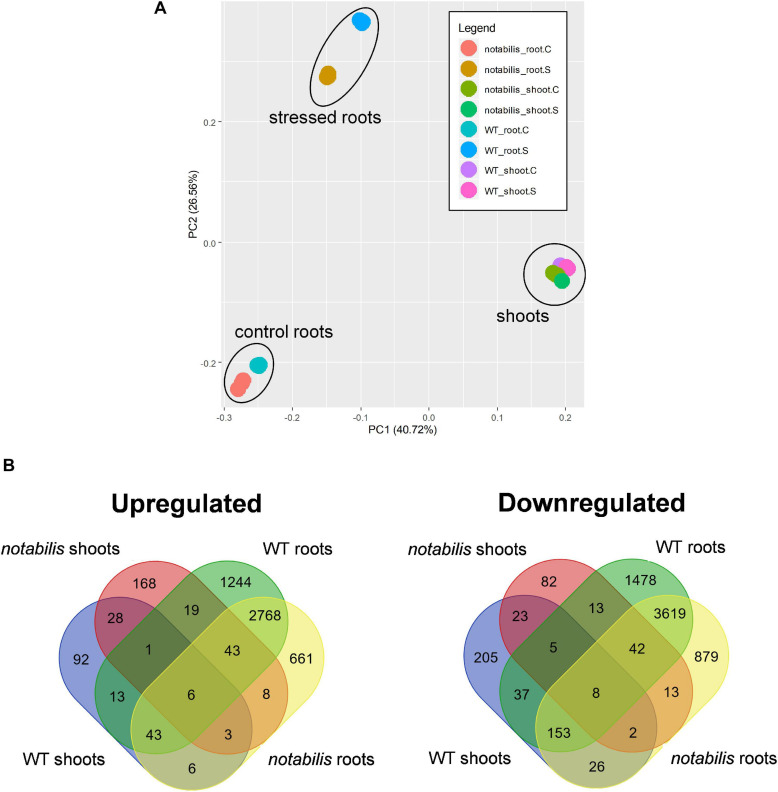
Principal component analysis of wild type (Lukullus) and *notabilis* gene expression profiles **(A)** and Venn diagrams of differentially expressed genes in response to soil waterlogging (*N* = 3) **(B)**.

In both Luk and *not*, soil flooding had a deeper impact on the transcriptional profiles of roots than shoots (Figure 2A): 9,492 DEGs (5,355 upregulated and 4,137 downregulated) and 8,280 DEGs (4,742 upregulated and 3,538 downregulated) for Luk (from now on, WT) and *not*, whereas these numbers went down to 651 (459 upregulated and 192 downregulated) and 652 (376 upregulated and 276 downregulated) in shoots of each of the two genotypes, respectively (Figure 2 and Supplementary Files S1, S2). This different impact correlated with the results from the PCA analysis, where PC1 and PC2 explained 40.7 and 26.6% of the variability, respectively. The transcript profiles showed little overlapping between tissues, and only 203 upregulated and 63 downregulated common transcripts were found between the roots and shoots of WT plants (3.5 and 1.4%, respectively), with similar numbers in *not*. The overlapping values between genotypes and within the same tissue were higher, especially for roots (6.8 and 37.5% for shoots and roots, respectively) (Figure 2B).

**FIGURE 3:**
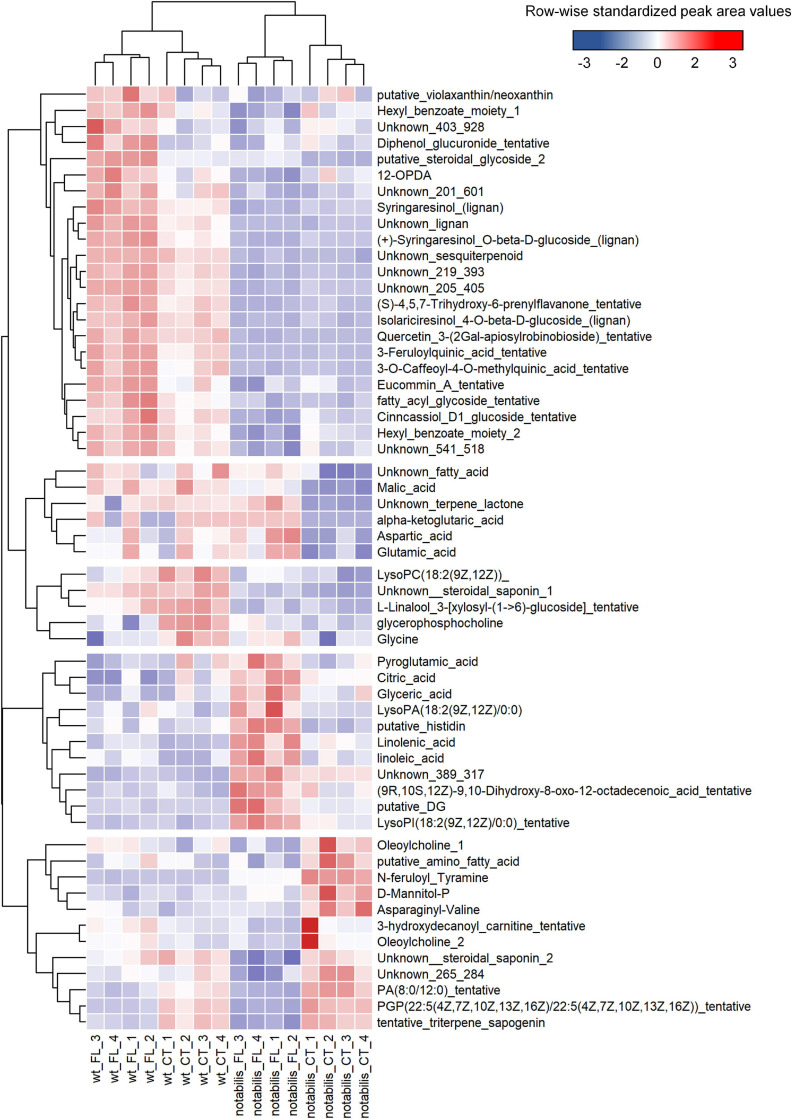
Heat map of differentially accumulated metabolites in shoots of wild type (Lukullus) and *notabilis* in response to soil flooding (*N* = 4). Data are normalized peak area values, row-wise standardized for representation.

**FIGURE 4:**
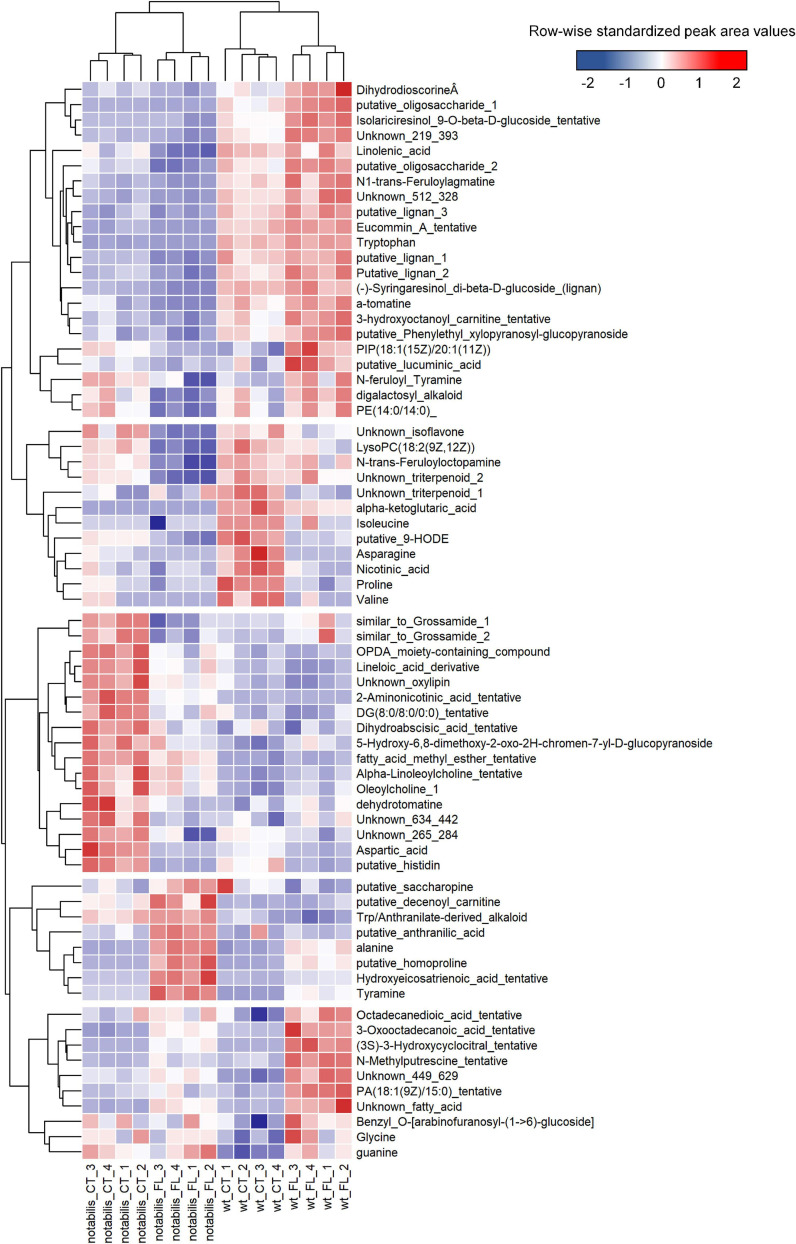
Heat map of differentially accumulated metabolites in roots of wild type (Lukullus) and *notabilis* in response to soil flooding (*N* = 4). Data are normalized peak area values, row-wise standardized for representation.

At the metabolic level, the impact of soil flooding was more pronounced in *not* than in WT (with 85 and 83 *vs.* 65 and 51 altered metabolites in shoots and root, respectively). Variations in metabolite levels were larger in roots, either by upregulation or downregulation (Supplementary File S3 and Supplementary Figure S4). There were also metabolites associated specifically to stress and to the combination of stress × genotype (Figures 3, 4). The number of metabolites whose variation pattern was compatible with stress induction was significantly lower in *not* than in WT in both leaves and roots. In the leaves of WT, this fraction was primarily composed of lignans, phenylpropanoids, oxylipins and other fatty acids, and lipid molecules, whereas the induced fraction in *not* leaves was particularly rich in fatty acids, phospholipids, oxylipins, and other lipid molecules. In roots, metabolites altered by flooding in WT primarily comprised, as in leaves, lignans, phenylpropanoids and derivatives, phospholipids, and different glycoalkaloids such as α-tomatine. The array of flooding-induced metabolites in *not* was again different from that of WT, including mostly amino acids (saccharopine, homoproline, and Ala), anthranilic acid, and related molecules (Figure 4). Moreover, metabolites whose levels were reduced in response to soil flooding were identified in shoots and roots. In shoots, the levels of amino acid Gly and two phosphocholine derivatives were reduced by soil flooding in WT, whereas mannitol phosphate, two metabolites annotated as oleoylcholine, feruloyl tyramine, a dipeptide, and different lipid substances (fatty acids, terpenoids, and carnitine–lipid conjugates) also showed higher levels in leaves of *not* control plants and were specifically reduced in response to soil flooding (Figure 3). Moreover, there were several metabolites comprising mostly phospholipids whose levels reduced in response to soil flooding irrespective of the ABA deficiency (Figure 3). Similarly, in roots, soil flooding also reduced a series of metabolites with higher levels in the roots of control WT plants: the amino acids Pro, Val, Ile, and Asn along with a 9-hydroxyoctadecadienoic, a phosphatidyl inositol phospholipid, and the TCA intermediate α-ketoglutaric acid. In *not*, a high number of metabolites that were specifically reduced by soil flooding showed also higher basal levels with respect to WT and included mostly fatty acids, oxylipins, two tentatively annotated lignans, the arylbenzofuran flavonoid grossamide, the amino acids Asp and His, feruloyl putrescine, dihydroabscisic acid, and the tomato glycoalkaloid dehydrotomatine (Figure 4).

### Characterization of the ABA Signaling Pathway in Roots of Flooded Tomato Plants

**FIGURE 5:**
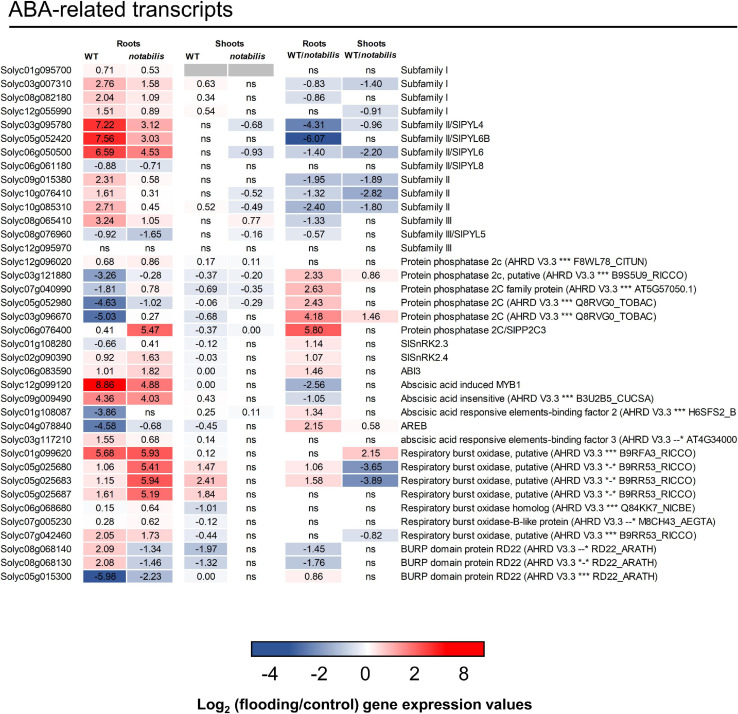
Expression of ABA signaling pathway-related transcripts in the shoots and roots of wild type (Lukullus) and *notabilis* in response to soil flooding. Data are mean values of log_2_-transformed flooded-to-control expression values (*N* = 3). Statistical significance was assessed after trimmed mean of *M* value-normalized data of genes meet false discovery rate-corrected *p* ≤ 0.05.

The transcript levels of key elements of the ABA signaling pathway were studied in the roots and shoots of WT and *not*, focusing on PYR/PYL/RCAR ABA receptors, PP2CA phosphatases, SnRK2 kinases, and downstream responses: ABA-dependent transcription factors, RBOHDs, and some LEAs (Figure 5). At least 15 genes encoding for different PYR/PYL/RCAR ABA receptors have been characterized in the tomato genome (Gonzalez-Guzman et al., 2014), of which 12 (three of subfamily I, three of subfamily III, and six of subfamily II) have been identified as DEGs in the RNAseq analysis (Figure 5 and Supplementary Files S1, S2). All identified ABA receptors in the tomato transcriptome except Solyc06g061180 (SlPYL8, subfamily II) and Solyc08g076960 (SlPYL5, subfamily III) exhibited an increased expression in response to soil flooding, which was more pronounced in WT than in *not* and restricted to the roots (Figure 5). The most prominent response in terms of fold change overexpression was observed in Solyc03g095780 (SlPYL4), Solyc05g052420 (SlPYL6B), and Solyc06g050500 (SlPYL6) belonging to subfamily II. Moreover, gene expression of clade A protein phosphatases 2C, involved in ABA signaling, was downregulated by soil flooding following the same trend as that of ABA levels but more intense in WT than in *not* plants. Interestingly, Solyc06g076400 (SlPP2C3) was strongly upregulated in *not* (a log_2_ fold change of 5.5 over controls *vs*. 0.41 in WT). Only two SnRK2s orthologs could be identified in the tomato transcriptome, SlSnRK2.3/Solyc01g108280 (AtSnRK2.6) and SlSnRK2.4/Solyc02g090390 (AtSnRK2.2/2.3) (Sun et al., 2011), which did not show any prominent transcriptional variation in response to soil flooding, except for Solyc02g090390 that showed a 1.6-log_2_ fold increase in *not*. Seven RBOHDs were identified in the tomato transcriptome. These are membrane-bound NADPH oxidases reported to be induced by ABA (Osakabe et al., 2014; Pilati et al., 2017) and involved in ROS production, particularly during hypoxia, constituting a putative key component of ROS/hormonal crosstalk signaling (Sasidharan et al., 2018). Five identified RBOHDs exhibited a different transcriptional response between WT and *not* compatible with the regulation of ABA signaling. Interestingly, the inability for full ABA synthesis resulted in increased RBOHD expression (Figure 5). In addition, five genes annotated as ABA-dependent transcription factors were identified in the RNAseq analysis, of which Solyc12g099120, encoding a MYB1 ABA-dependent transcription factor whose expression has been linked to ABA signaling (Abuqamar et al., 2009), and Solyc03g117210, encoding an AREB/ABF (ABA-responsive element binding factor) transcription factor, were induced in waterlogged tissues, showing a higher fold change in WT. Solyc09g009490 encoding an ABA insensitive-like transcription factor was also induced by soil flooding to an almost identical extent in WT and *not*. Two genes—Solyc01g108087 and Solyc04g078840—both encoding AREB/ABF transcription factors, were downregulated by soil flooding, but to a much higher extent in WT. Finally, a group of genes whose expression is known to be ABA-dependent showed a different response to soil flooding and also between genotypes. Two tomato RD22-like genes, Solyc08g068140 and Solyc08g068130, were upregulated in WT and downregulated in *not*, whereas Solyc05015300 and Solyc04g078840, another RD22-like gene and a RB29A-like gene, respectively, were repressed in response to soil flooding, but more prominently in WT than in *not* (Figure 5). In shoots, the transcriptional response of ABA-related transcripts to soil flooding was much more prominent in WT than in *not*, in which most genes rendered non-significant results (Figure 5).

### The Ethylene and N-degron Pathway in Roots of Flooded Tomato Plants

**FIGURE 6:**
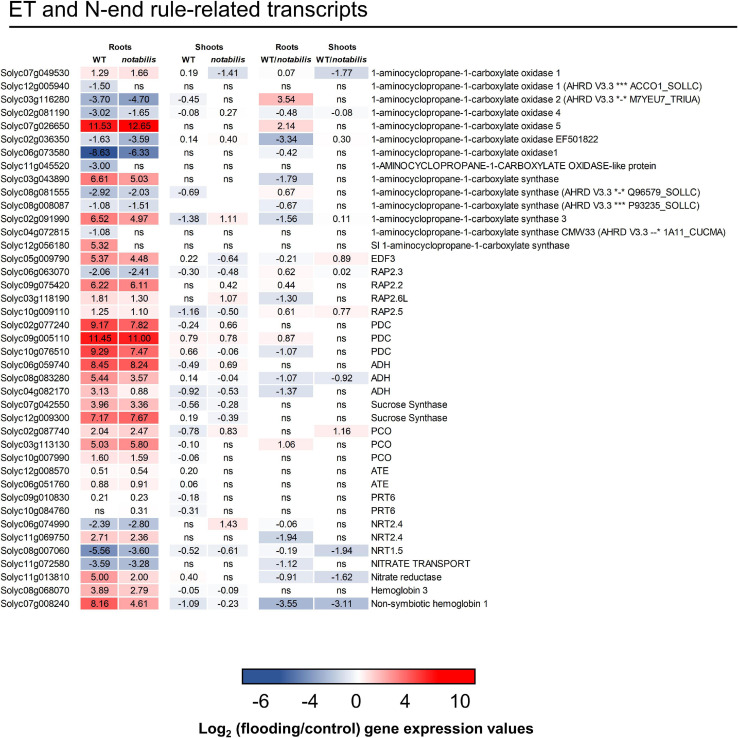
Expression of ethylene signaling and N-end rule pathway-related transcripts in shoots and roots of wild type (Lukullus) and *notabilis* in response to soil flooding. Data are mean values of log_2_-transformed flooded-to-control expression values (*N* = 3). Statistical significance was assessed after trimmed mean of *M* value-normalized data of genes meet false discovery rate-corrected *p* ≤ 0.05.

Ethylene plays a pivotal role in the hypoxic response inducing the expression of ERFVIIs that induce adaptive responses to hypoxia. In the presence of O_2_, these transcription factors are degraded through the N-end rule pathway (Gibbs et al., 2011; Voesenek and Sasidharan, 2013). Under the conditions assayed in this work, some genes coding for ACC synthases and ACC oxidases were highly upregulated in roots of both genotypes (Figure 6). Moreover, genes previously annotated as ERFVIIs in the tomato transcriptome (Safavi-Rizi et al., 2020) were upregulated in response to soil flooding in roots as well as typical hypoxia responses, such as the induction of pyruvate decarboxylase (Solyc02g077240, Solyc09g005110, and Solyc10g076510), alcohol dehydrogenase (Solyc06g059740, Solyc08g083280, and Solyc04g082170), and sucrose synthase (Solyc07g042550 and Solyc12g009300) genes involved in the metabolic shift under hypoxic conditions (Lee et al., 2011), confirming the activation of ET/O_2_-mediated hypoxia response (Figure 6). In shoots, on the contrary, no clear transcriptional response was observed. Genes encoding key steps of the proteolytic N-end rule pathway, such as plant cysteine oxidases (PCOs, Solyc02g087740, Solyc03g113130, and Solyc10g007990), ATE1/2 (Solyc12g008570 and Solyc06g051760), and PROTEOLYSIS6 (PRT6) orthologs (Solyc09g010830 and Solyc10g084760), showed a similar upregulation in both genotypes. Moreover, genes involved in NO biosynthesis and scavenging (Solyc08g068070 and Solyc07g008240 orthologs of hemoglobin 3 and phytoglobin 1, respectively) were upregulated in response to soil flooding in roots of WT to a higher extent than in *not* (Figure 6), suggesting that NO could be produced under the conditions assayed in this work.

### The Metabolic Response of Flooded *vs*. Aerated Tomato Tissues

**FIGURE 7:**
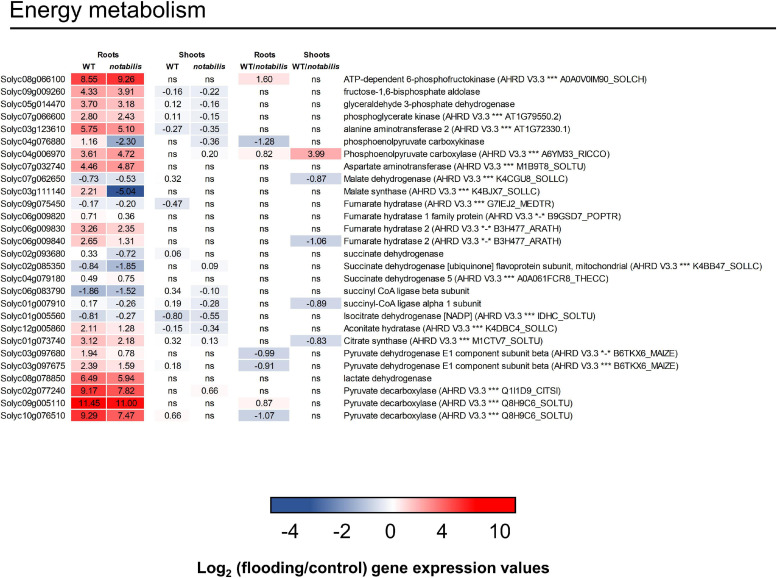
Expression of energy metabolism-related transcripts (glycolysis and tricarboxylic acid cycle) in the shoots and roots of wild type (Lukullus) and *notabilis* in response to soil flooding. Data are mean values of log_2_-transformed flooded-to-control expression values (*N* = 3). Statistical significance was assessed after trimmed mean of *M* value-normalized data of genes meet false discovery rate-corrected *p* ≤ 0.05.

The shift to an anaerobic metabolism in roots, as a primary response in hypoxic tissues, was investigated in more detail by analyzing the expression of relevant genes in the tomato transcriptome and the accumulation of key metabolites (Figure 7). Transcript data supported the activation of the glycolytic and fermentative pathways (pyruvate decarboxylases and lactate dehydrogenase) and the activation of Ala aminotransferase which was accompanied by a strong accumulation of Ala (Figures 4, 7). Along with the induction of the fermentative pathway, the expression of genes involved in key steps of the TCA cycle was either downregulated (isocitrate dehydrogenase, succinyl CoA synthetase, succinate dehydrogenase, or malate dehydrogenase) or showed a slight upregulation (citrate synthase, aconitase, or fumarase). Conversely, Asp aminotransferase, involved in the anaplerotic refilling of oxalacetate from Asp, showed an upregulation in both genotypes, resulting in severe depletion of the amino acid. Genes involved in other anaplerotic pathways such as malate synthase and phosphoenol pyruvate carboxykinase showed not only a moderate upregulation in WT but also a strong downregulation in *not*. At the metabolic level, the accumulation of succinate was observed, whereas malate was depleted with no differences that could be associated to ABA deficiency (Figures 3, 4). Moreover, a clear accumulation of α-ketoglutaric acid, Asp, citric acid, fumaric acid, and malic acid levels was observed in shoots.

### Analysis of Genes Potentially Regulated by ABA in Flooded Roots of Tomato Plants

**FIGURE 8:**
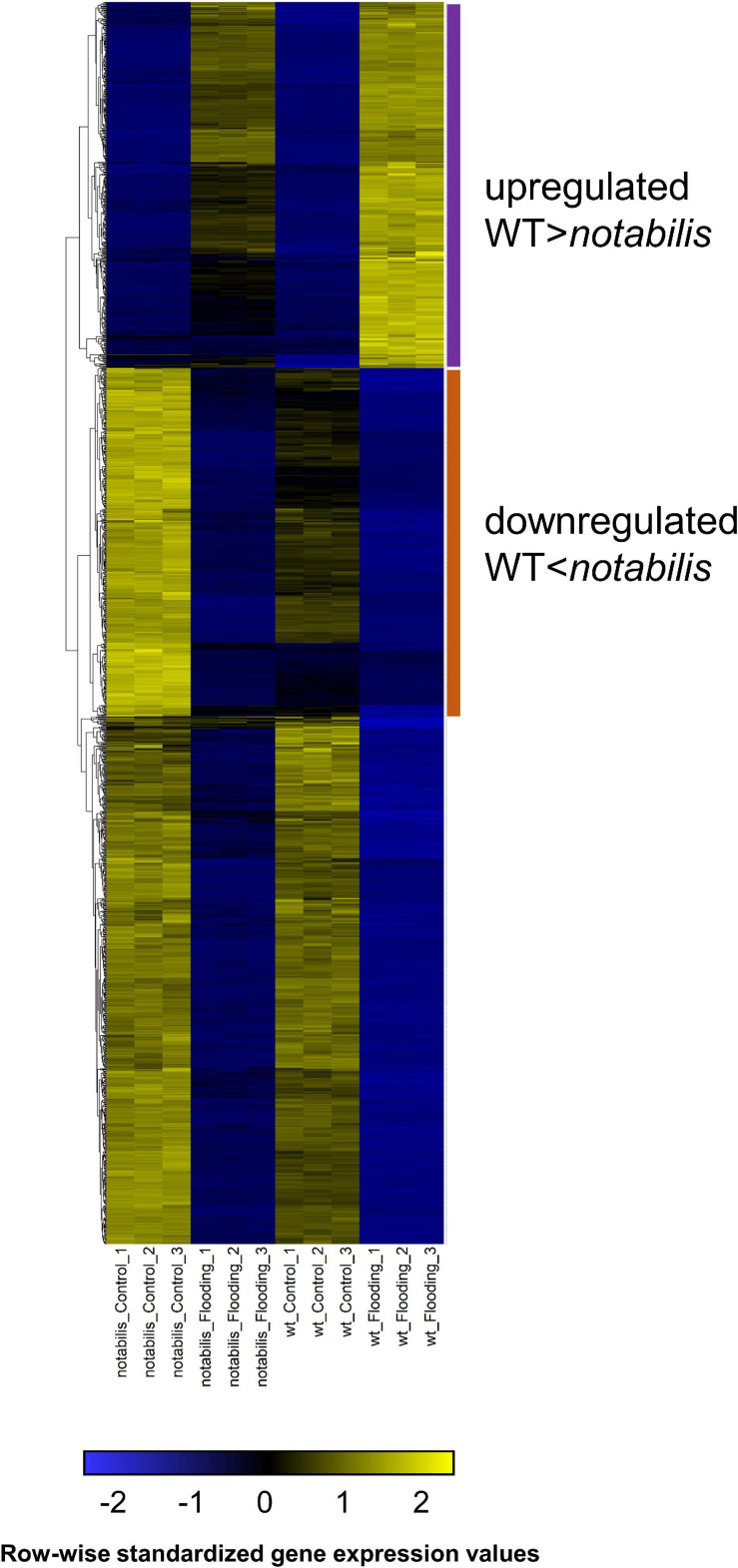
Expression pattern of differentially expressed genes in roots of wild type (Lukullus) and *notabilis* in response to soil flooding (*N* = 3). Data are in fragments per kilobase of transcript per million mapped reads values. Upregulated genes are labeled in magenta; downregulated genes are labeled in orange.

The genome-wide expression analysis showed that variation in transcript number was more intense in flooded than in aerated organs, in line with the more intense metabolic response in these organs (Supplementary File S3 and Figure 4). Therefore, subsequent analyses were focused on the root transcriptome and aimed to identify gene clusters with a more intense transcriptional response (either upregulation or downregulation) in WT than in *not*. Therefore, FPKM values were subjected to HCA and represented as a heat map for better visualization (Figure 8). From the representation, two interesting trend clusters could be observed: (i) upregulated by soil flooding, comprising genes with increased gene expression upon imposition of soil flooding and had higher fold change values in WT than in *not* (Figure 8, magenta) and (ii) downregulated by soil flooding, including genes with reduced gene expression upon imposition of soil flooding and having lower fold change values in WT than in *not* (Figure 8, orange). In both clusters, the expression trends showed a negative correlation with ABA levels in roots in flooded and non-flooded plants, compatible with the hypothesis that ABA depletion acts as a positive hormonal signal in flooded plant organs by enhancing or attenuating gene fold change upregulation or downregulation, respectively.

This hypothesis was further investigated by screening promoters of genes included within these clusters for the presence of known *cis*-regulatory elements (Tables 1, 2). This analysis rendered several hexamer motifs with a significant enrichment in both upregulated and downregulated fractions. Interestingly, promoters of the genes from the upregulated gene cluster showed enrichment in abscisic acid-responsive elements (ABRE) ACGT sequences, with values ranging between 1.18 and 1.25; this is 18–25% probability above the expected distribution in the tomato genome (Table 1). Interestingly, other *cis*-elements not involved in ABA signaling have been identified as enriched in the upregulated gene fraction. For instance, a G-box CACGTG sequence, known to be a binding site for MYC2 in *Arabidopsis thaliana*, showed 35.6% enrichment in the upregulated fraction. Moreover, a W-box GTCATA, known as a binding recognition site for WRKY3 and WRKY6 in *A. thaliana*, showed 14% enrichment. Other identified binding sites were those sharing CACT tetranucleotide sequences found in the *cis*-regulatory element in the distal region of the phosphoenol pyruvate carboxylase gene of *Flaveria trinervia*, an annual weed from the Asteraceae family, and the AATATT found in promoters of the rolD gene from *Agrobacterium rhizogenes*, which are root-specific. Promoters from genes included in the downregulated gene fraction showed a significant enrichment in GGCCCA motif (52% above the expected genome distribution), which is abundant in promoters of nuclear genes encoding components of the oxidative phosphorylation machinery (Table 2). Other overrepresented motifs are AGTACT (28–34%), present in promoters of copper-responsive genes in *Chlamydomonas reinhardtii* and involved in their oxygen response, followed by ACGT motifs or A-box found in the octopine synthase gene (28.6%) and ACGT desiccation responsive (21.7%). Those with much lower enrichment values were the CACT tetranucleotide sequences mentioned above, CTAATA involved in cytokinin signaling, and CAAT promoter consensus sequence found in legA gene from *Pisum sativum*, a storage protein.

**TABLE 1:** Known transcription factor binding motifs enriched in promoters (∼1,000 bp upstream of the transcription start site) of genes upregulated in flooded roots showing a higher expression in wild type than in *notabilis* (Figure 8).

Motif	# In genome promoters	# Total genome promoters	# In promoters upregulated genes	# Total in promoters upregulated genes	Enrichment	*p-value* (FDR-corrected)	Known binding activity
**CACGTG**	3824	34417	128	849	1.35692989	0.000215	G box/binding site for AtMYC2
**CACGTA**	6883	34417	212	849	1.24860024	0.0002044	ABRE
**ACGTAT**	8196	34417	247	849	1.22168805	0.000198	ACGT/desiccation responsive
**ACGTAA**	9370	34417	280	849	1.21138938	0.00010403	ACGT/desiccation responsive
**ACGTGA**	7661	34417	224	849	1.18529889	0.00229434	ACGT/desiccation responsive
**GTCATA**	13259	34417	373	849	1.14041621	0.0006356	W box/binding site for WRKY3 and WRKY6
**ATTATC**	21924	34417	600	849	1.10942201	8.42E-06	Consensus GT-1 binding site in many light-regulated genes
**ATAGTA**	20635	34417	564	849	1.10800049	4.79E-05	Tetranucleotide (CACT) is a key component of Mem1 (mesophyll expression module 1) found in the *cis*-regulatory element in the distal region of the phosphoenolpyruvate carboxylase (ppcA1) of the C4 dicot *F. trinervia*
**AATACT**	21847	34417	596	849	1.10590997	1.74E-05	Tetranucleotide (CACT) is a key component of Mem1 (mesophyll expression module 1) found in the cis-regulatory element in the distal region of the phosphoenolpyruvate carboxylase (ppcA1) of the C4 dicot *F. trinervia*
**AATATT**	27066	34417	736	849	1.10234886	6.94E-10	Motif found both in promoters of rolD

**TABLE 2:** Known transcription factor binding motifs enriched in promoters (∼1,000 bp upstream of the transcription start site) of genes downregulated in flooded roots showing a lower expression in wild type than in *notabilis* (Figure 8).

Motif	# In genome promoters	# Total genome promoters	# In promoters downregulated genes	# Total in promoters downregulated genes	Enrichment	*p*-value (FDR-corrected)	Known binding activity
**GGCCCA**	3947	34417	110	631	1.52008968	5.05E-06	“Site II element” found in the promoter regions of cytochrome genes (Cytc-1, Cytc-2) in *Arabidopsis*; overrepresented in the promoters of nuclear genes encoding components of the oxidative phosphorylation (OxPhos) machinery from both *Arabidopsis* and rice
**AGTACT**	6900	34417	170	631	1.34382737	1.53E-05	GTAC is the core of a CuRE (copper-response element) found in Cyc6 and Cpx1 genes in *Chlamydomonas*; also involved in oxygen response of these genes
**TACGTA**	5002	34417	118	631	1.28671384	0.00216669	“A-box” according to the nomenclature of ACGT elements by Foster et al. (1994) [FASEB J, 8:192–200]; one of ACGT elements; found in ocs gene
**GTACTA**	10009	34417	235	631	1.28062161	5.08E-06	GTAC is the core of a CuRE (copper-response element) found in Cyc6 and Cpx1 genes in *Chlamydomonas*; also involved in oxygen response of these genes
**ACGTAA**	9370	34417	209	631	1.2166071	0.00056655	ACGT/desiccation responsive
**GTAGTA**	11059	34417	238	631	1.17382878	0.00156573	Tetranucleotide (CACT) is a key component of Mem1 (mesophyll expression module 1) found in the *cis*-regulatory element in the distal region of the phosphoenolpyruvate carboxylase (ppcA1) of the C4 dicot *F. trinervia*
**CTAATA**	20787	34417	435	631	1.14140848	4.31E-06	The sequence critical for cytokinin-enhanced protein binding *in vitro*, found in -490 to -340 of the promoter of the cucumber (CS) POR (NADPH-protochlorophyllide reductase) gene
**TAAGTA**	19684	34417	409	631	1.13332274	4.80E-05	Tetranucleotide (CACT) is a key component of Mem1 (mesophyll expression module 1) found in the *cis*-regulatory element in the distal region of the phosphoenolpyruvate carboxylase (ppcA1) of the C4 dicot *F. trinervia*
**ATAGTA**	20635	34417	426	631	1.12602693	4.54E-05	Tetranucleotide (CACT) is a key component of Mem1 (mesophyll expression module 1) found in the cis-regulatory element in the distal region of the phosphoenolpyruvate carboxylase (ppcA1) of the C4 dicot *F. trinervia*
**CAATTA**	23958	34417	484	631	1.10189054	3.82E-05	“CAAT promoter consensus sequence” found in legA gene of pea
**ACAATA**	24128	34417	487	631	1.10090866	3.65E-05	“CAAT promoter consensus sequence” found in legA gene of pea

Following promoter analysis, the genes in each gene cluster showing *cis*-elements in their promoters associated to ABA and/or JA signaling and dependent expression were extracted and annotated. No GO enrichment could be found in the upregulated fraction, but a relatively high number of genes could be directly associated to (i) ABA-dependent responses (protein phosphatases 2C, Solyc01g080400, and Solyc07g064310; the ABI five binding protein 2 (AFP2), Solyc02g088910; an ABA-dependent MYB transcription factor, Solyc12g099120; an early dehydration responsive 1 ortholog ERD1, Solyc07g048110 and ABA receptors, Solyc06g050500, and Solyc08g065410), (ii) JA-dependent responses (a JAZ protein, Solyc07g042170; the TOPLESS co-repressor, Solyc03g117360; wound-responsive proteins related to JA signaling, Solyc07g054770 and Solyc07g054790; the defense protein patatin, Solyc10g080690, a phospholipase D, Solyc10g024370, and a lipoxygenase involved in JA biosynthesis, Solyc01g099200). In addition, a great number of genes could be grouped in a “stress signaling” cluster, including transcription factors such as WRKYs, Solyc02g080890, Solyc05g012500, Solyc05g015850, Solyc10g011910, and Solyc12g011200; NAC, Solyc02g088180; MYB transcription factor, Solyc09g091880, Solyc04g014470; different bHLH transcription factors, Solyc06g069370, Solyc07g052670 and Solyc09g098110; two R2R3MYB transcription factors, Solyc04g014470 and Solyc03g116100; bZIP transcription factors, Solyc01g079480 and Solyc04g005170; a C_2_H_2_-like zinc finger protein, Solyc02g079920; and an AP2/B3 transcription factor, Solyc11g066630. Moreover, other genes coding for stress-related processes are such as a protein containing the jumonji domain, involved in histone demethylation, regulation of gene expression, and stress responses, Solyc08g081000; an E3 SUMO-protein ligase, Solyc01g095130, involved in drought stress responses; a tonoplast intrinsic protein (aquaporin)-encoding gene, Solyc06g074820; a dehydration-induced protein Solyc06g071780; a gene encoding Argonaute 2a subunit involved in miRNA processing, Solyc02g069260. Other signaling pathways that were represented in this group of genes were GA signaling (Solyc07g063940, Solyc11g017440, and Solyc05g054170), auxin signaling (Solyc03g082520 and Solyc10g011660), and ethylene and oxygen signaling (Solyc02g077370, Solyc09g009970, Solyc12g096220, Solyc03g113130, Solyc08g077230, and Solyc07g042550).

Included in this upregulated cluster, different genes involved in primary and, particularly, secondary metabolism appear to have ABA and JA-dependent transcription factor recognition motifs (Supplementary File S4). Within the primary metabolism, identified genes are related to energy metabolism and usage of amino acid carbon skeleton as energy sources. Among genes related to secondary metabolism are those encoding for phytoene synthase 1, sesquiterpene synthase, and carotenoid cleavage dioxygenase (Solyc03g031860, Solyc07g052120, and Solyc08g066720) and phenylalanine ammonia-lyase (Solyc02g090500 and Solyc05g056170), which are key in regulating the metabolic flow when sesquiterpenoid and phenylpropanoid biosynthesis arises. Moreover, different genes involved in the modification of phenylpropanic acids for lignin biosynthesis (Solyc06g035960, Solyc06g082530, Solyc12g042460, Solyc01g107910, Solyc02g093270, and Solyc10g005060) along with genes encoding proteins directly involved in the modification of secondary cell wall components (Solyc08g075020, Solyc08g005610, Solyc11g031950, Solyc01g091050, and Solyc01g079950) occurred within this group. In addition, several genes encoding phospholipases D and C, involved in the processing of membrane phospholipids to generate different lipid-derived signaling compounds, were identified (Solyc08g080130, Solyc06g007120, and Solyc06g082000). Finally, a gene encoding dihydroflavonol 4-reductase-like protein (Solyc12g005350), involved in the synthesis of anthocyanidins from flavonols, was also identified among the list of genes whose promoters include ABREs and W-box or G-box motifs (Supplementary File S4).

As in the upregulated cluster, no enriched GO terms could be found in the list of downregulated genes (Table 2 and Supplementary File S5), but several genes could be associated to metabolic and or signaling roles. In terms of hormone signaling, several genes annotated as auxin-responsive were identified (Solyc09g056360, Solyc08g079150, Solyc10g006610, Solyc07g009330, and Solyc07g014620), along with a gene encoding a 12-oxophytodienate reductase 3 involved in JA biosynthesis (Solyc07g007870), ethylene signaling (Solyc02g081190, encoding a 1-aminocyclopropane-1-carboxylate oxidase 4 involved in ethylene biosynthesis), and a serine/threonine-protein kinase (Solyc07g053080) potentially involved in ABA signaling. Only two genes were annotated within the stress signaling category (Solyc04g005100 and Solyc05g007110, encoding a MYB and a WRKY76 transcription factor, respectively). Among the genes included in the primary metabolism category, genes involved in energy metabolism (Solyc01g090610, Solyc01g108860, Solyc06g083790, Solyc10g005620, Solyc09g009520, Solyc12g098660, and Solyc08g068400) were the most prominent. Other minor genes included those involved in iron assimilation (Solyc01g094890) and photorespiration (Solyc10g007600). The secondary metabolism category included genes mostly involved in primary and secondary cell wall modification and extension (Solyc07g043390, Solyc07g017600, Solyc09g091430, Solyc08g060970, Solyc07g064870, Solyc12g014420, Solyc05g012380, Solyc03g093390, and Solyc06g005560). Other genes identified were Solyc03g097030 and Solyc07g005760, involved in lignin biosynthesis, and Solyc01g091330, encoding a glutathione S-transferase involved in the detoxification of lipid hydroperoxides, consistent with the downregulation of an ascorbate peroxidase gene within the same list (Solyc06g005160).

## Discussion

In this work, the role of ABA on the regulation of genetic and metabolic responses to soil flooding was investigated. In previous works carried out in citrus, it was shown that prolonged soil flooding causes an overreduction of endogenous ABA levels in waterlogged roots at the expense of catabolism and hexose conjugation (Arbona and Gómez-Cadenas, 2008; Arbona et al., 2017). Moreover, it has been shown that soil flooding induces a rearrangement of the secondary metabolism in submerged tissues in citrus, mainly influencing the biosynthesis of phenylpropanoids and lignin precursors, as observed in citrus (Argamasilla et al., 2013) and soybean (Wang et al., 2017; Coutinho et al., 2018).

The data presented in Figure 1 regarding physiological and hormonal response agree with previous results obtained in citrus (Arbona and Gómez-Cadenas, 2008; Arbona et al., 2009, 2017), but, interestingly, no significant variation in foliar ABA levels could be observed despite the reduction in *E* and *g*_*s*_ recorded in WT. The upregulation of RBOHD gene expression observed in leaves of flooded WT could be partially responsible for stomatal closure through an active ROS signaling in shoots (Yeung et al., 2018). Continuous waterlogging for 24 h was sufficient to induce ABA depletion in roots before any foliar accumulation of the plant hormone could be detected, therefore constituting a convenient experimental system to study early responses to soil flooding. According to transcript and metabolite profiling data, the effects of 24 h of soil flooding were more pronounced in roots than in shoots, as shown by the principal component analysis (Figure 2A). As expected, *not* exhibited lower ABA levels (50 and 89% lower than those found in the leaves and roots of WT, respectively), resulting in an enhancement of transpiration, an impairment in stomatal response to soil flooding, and a reduction in LWP, although this had no apparent correlation with leaf RWC (Figure 1). Interestingly, despite the low ABA levels found in roots of *not*, soil waterlogging reduced NCED (Solyc07g056570) expression to a higher extent than in WT, which correlates with the reduction of ABA hormone levels observed in *not* roots (Figure 1B). The expression of different genes involved in ABA signaling was induced in the roots of flooded plants and, generally, to a higher extent in WT than in *not*. Most genes encoding PYR/PYL/RCAR ABA receptors were upregulated by soil flooding as in Arbona et al. (2017). The expression of all genes (except Solyc06g076400) encoding protein phosphatases 2C was likewise repressed in WT. Moreover, the expression levels of all these genes were higher in control WT than in *not*, in agreement with their negative regulatory role in ABA signaling and the low hormone levels present in *not*, especially, in flooded roots (Figure 5). These results altogether suggested that ABA signaling was operative in flooded roots despite the low ABA levels, indicating that the signaling machinery adapts to the actual ABA levels constituting a potential soil flooding-specific signal. To further support this hypothesis, it was previously reported in citrus and *Arabidopsis* that some PYR/PYL/RCAR ABA receptors inhibit PP2C activity to a great extent even in the presence of very low amounts of ABA (Arbona et al., 2017; Tischer et al., 2017), probably as a result of their higher affinity for the ABA–PP2C complex. Therefore, the combination of higher ABA receptor levels along with their reported higher affinity for the hormone would enable a higher efficiency to inhibit PP2C activity, ensuring an active signal transduction to develop the genetic and metabolic response. Moreover, the expression of a number of certain ABA-dependent transcription factors, particularly Solyc12g099120 encoding an ABA-dependent MYB1, Solyc03g117210, encoding an AREB3 tomato ortholog, and two RD22 orthologs (Solyc08g068140 and Solyc08g068130), was induced (Figure 5), confirming ABA signaling under these conditions. Interestingly, genes encoding RBOHDs showed a clear upregulation in *not* despite their reported dependence on ABA signaling, which might account for an improved recovery after waterlogging as previously reported in *Arabidopsis* (Yeung et al., 2018).

The expression of genes involved in the ET/O_2_ signaling pathway did not differ greatly between flooded tissues in WT and *not*. Only ACC synthase-encoding genes had a slightly higher expression in WT than in *not*. From the identified ERFVII tomato orthologs, Solyc09g075420, a RAP2.2 ortholog, showed the most prominent expression. Moreover, all reported hypoxia survival responses (PDC, ADH, and sucrose synthase) showed a genetic activation in flooded tissues with no significant differences between genotypes, altogether indicating that ET/O_2_ signaling was active in the roots of flooded tomato plants. Genes encoding plant cysteine oxidases (Solyc02g087740, Solyc03g113130, and Solyc10g007990) showed a significant transcriptional activation with no differences between genotypes, suggesting the activation of the N-degron pathway (Figure 6). It has been recently suggested that tomato ERFVIIs might be uncoupled from hypoxia-responsive gene induction (Hartman et al., 2020); nevertheless, our data indicate a clear correlation between the RAP2.2 ortholog and the expression of hypoxia-related transcripts. Interestingly, genes encoding known NO synthesis genes in plants, Solyc11g013810, encoding a nitrate reductase, and genes encoding for phytoglobins (Solyc08g068070 and Solyc07g008240), known for their ability to scavenge NO (Gupta et al., 2019), showed an upregulation in flooded tissues, with a larger fold change value in WT than in *not*. This indicates that ABA influences NO biosynthesis and efficient scavenging, subsequently affecting its regulatory role under hypoxia, as already reported in *Arabidopsis* and *Lotus japonicus* (Rubio et al., 2019), and suggesting that ABA could potentially act as a modulator of the plant N-degron pathway through modulation of NO levels to fine-tune the ET/O_2_ signaling pathway. Moreover, jasmonate (JA and JA-Ile) levels were also affected by soil waterlogging in the same way as that of ABA, as already reported in citrus (Arbona and Gómez-Cadenas, 2008; Arbona et al., 2017), and correlated with the repression of genes encoding for allene oxide synthase and cyclase (Supplementary Figure S5). This reduction of jasmonate levels was correlated with the upregulation of several genes encoding for jasmonate ZIM domain proteins (JAZ) with no differences between genotypes. Moreover, genes encoding for defensin-like proteins, known to be induced by jasmonates (Balbi and Devoto, 2008; Vives-Peris et al., 2017), were also upregulated by soil flooding (Supplementary Figure S5). In all cases, the relevant transcriptional response was observed only in hypoxic tissues whereas aboveground aerated tissues showed either a significantly reduced response or no response at all.

Metabolite profiles distinguished between genotypes (PC1) and, at a more particular level, tissues (PC2, Supplementary Figure S4). Nevertheless, analyzing tissues separately, the impact of soil flooding on shoot metabolites was higher in *not* than in WT in terms of the number of altered metabolites and fold change variation, whereas in roots the response was similar (Supplementary Figure S4). In general, the number of repressed metabolites was higher, especially in roots, and included lipid-derived molecules and amino acids as the most represented metabolite classes. In this work, most annotated lipid-derived molecules were PCs, LysoPCs, or PEs (Figures 3, 4), which showed an accumulation pattern similar to that of ABA and jasmonates and ABA and JA-dependent induction of genes involved in phospholipid cleavage to release lipid-derived signals that could act in JA or other signaling pathways correlated with the accumulation of the derived metabolites: essentially fatty acids and oxylipins. Levels of amino acids (except for Ala) decreased in response to soil flooding in both tissues, which could be associated to their requirement as suppliers for TCA. As expected, the metabolic switch from aerobic to anaerobic could be clearly observed at the transcriptional level in flooded tissues (Figure 7): first, the activation of the glycolytic pathway along with the fermentative reactions and the downregulation of genes involved in the TCA cycle (Figure 7). The upregulation of Ala and Asp aminotransferases is consistent with the upregulation of malate synthase, citrate synthase, aconitate hydratase, and fumarate hydratase, accounting for certain TCA functioning and the production of NADH. To this respect, Asp and Ala showed an opposite behavior in the roots and shoots, especially in *not* (Asp accumulated in shoots and depleted in roots, and Ala showed an opposite pattern). This could be associated to the requirement of Asp in hypoxic roots to feed the TCA with oxalacetate *via* the Asp aminotransferase anaplerotic pathway. In hypoxic tissues, the accumulation of Ala occurs at the expense of pyruvate through Ala aminotransferase, as supported by transcriptional and metabolic data (Figures 4, 7), subsequently linking glycolysis and the TCA cycle (Rocha et al., 2010). Following this mechanism, Asp and Glu accumulated in tomato aerial tissues could be basipetally transported to hypoxic roots and act as substrates for oxalacetate production in roots. According to data presented in this manuscript, the root basal levels of Asp in *not* were higher than in WT, suggesting that the accumulation of this metabolite is not restricted to stress conditions and ABA might directly or indirectly regulate their build-up. To this respect, a significant enrichment in ABA-related cis-acting elements was found in promoters of genes involved in energy metabolism and phenylpropanoid and sesquiterpenoid biosynthesis in the upregulated fraction of genes (Figure 8 and Table 1). Among the genes involved in energy metabolism, the identification of genes encoding proteins involved in Asp and Glu metabolism as well as acetyl-CoA, ATP, and starch biosynthesis is remarkable, suggesting the involvement of ABA and JA in the modulation of certain steps in the production of energy in hypoxic tissues, particularly the biosynthesis of Glu from 2-oxoglutarate or transamination from Gln and the production of oxalacetate from Asp *via* Asp oxidase and from Asp and α-ketoglutarate *via* Asp aminotransferase (Figures 3, 4 and Supplementary File S3). It is also interesting to note that, among the genes identified after promoter analysis, Glu formiminotransferase 1 (Solyc07g054430) appears along with dihydrofolate reductase (Solyc04g074950), suggesting its involvement in the catabolism of His as supported by metabolomics data (Figures 3, 4).

**FIGURE 9:**
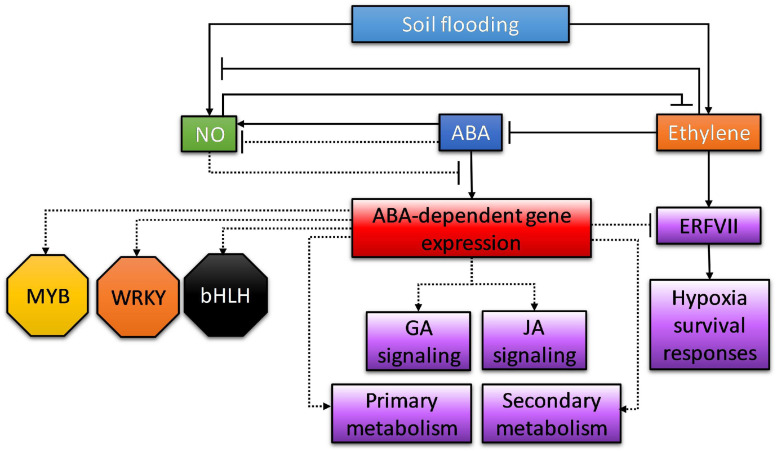
Summary of the roles of abscisic acid in waterlogged tissues, including its interaction with other signaling pathways. Solid lines indicate interactions that have already been confirmed in other works and experimental systems; dotted lines indicate interactions inferred from transcriptional and/or promoter analysis presented in this work.

Moreover, among the upregulated fraction, not only genes involved in ABA signaling, such as PYR/PYL/RCAR ABA receptors, were identified as harboring desiccation responsive or ABRE motifs in their promoters. Genes involved in JA, ethylene, O_2_, GA, or IAA signaling were also identified, pointing at a multifaceted crosstalk of ABA with other signaling pathways. Interestingly, orthologs of TOPLESS and NINJA family AFP2 were found as part of the JA signaling category harboring desiccation and ABRE motifs in their promoters. It has been described that ABA triggers the expression of AFP genes which, in turn, can bind to ABI5 in *Arabidopsis* seedlings. Moreover, it has also been described that AFPs may act in modulating ABA responses through TOPLESS-dependent chromatin modification (Lynch et al., 2017). To this respect, the TOPLESS protein interacts with NINJA acting as a transcriptional repressor in response to JA (Pauwels et al., 2010). Therefore, it is likely that, in tomato, both genes are induced by ABA and act as transcriptional regulators of JA-dependent genes, subsequently constituting potential effectors of the ABA-JA crosstalk under these conditions. As mentioned above, genes harboring *cis*-elements as ABRE, W-box, or G-box motifs in their promoters included some involved in ET signaling, such as ethylene response factor C.5 (Solyc02g077370) and a two-component response regulator (Solyc08g077230), and O_2_ signaling, such as plant cysteine oxidase (Solyc03g113130), sucrose synthase (Solyc07g042550), Rho GTPase-activating protein gacA (Solyc09g009970), and Rop guanine nucleotide exchange factor (Solyc12g096220), also suggesting that ABA and/or JA could act in modulating the plant O_2_ response. The presence of MYB2, WRKY, and bZIP binding motifs in genes co-expressed in response to hypoxia in *Arabidopsis* has already been reported, and the prevalence of AtMYB2 as an important regulator of genetic responses to low oxygen conditions was highlighted (Liu et al., 2005). To this respect, it has been recently shown that these transcription factors, along with bHLH and hypoxia-responsive elements, are the most relevant TF hubs involved in reprogramming the transcriptional response to soil flooding (Reynoso et al., 2019), although their relative participation can be highly flexible among different plant species. This is directly related to the variation in the presence of *cis*-acting binding sites in the promoters of co-expressed genes in response to flooding. Our results are consistent with these previous reports and expand the knowledge to an ABA-dependent subcircuitry which might also involve interactions with JA signaling. This network of interactions of ABA with other signaling and response pathways in waterlogged tissues is summarized in Figure 9.

In the list of downregulated genes, ABA-related *cis*-elements were not as overrepresented as in the upregulated list, and among the list of genes harboring these motifs, auxin signaling-related transcripts were found (Supplementary File S4). This is in agreement with their described role in response to soil flooding as growth-stimulating plant hormones in interaction with gibberellins (Cox et al., 2006) to regulate different plant responses such as adventitious root emergence and growth (Steffens et al., 2006).

Abscisic acid is acknowledged as the key regulator of plants’ adaptive responses to challenging environmental conditions, namely, drought, salinity, or high temperatures, but its involvement in responses to soil flooding is less well known. The work presented here shows that ABA deficiency alters water relations and genetic and metabolic reprogramming of shoots and roots to short-term soil flooding in tomato, a plant species that develops specific adaptations to soil hypoxia (Vidoz et al., 2016). Soil flooding induces ABA catabolism in hypoxic tissues, reducing active hormone levels which, in turn, may act as a specific signal to this adverse condition altering transcript and metabolite profiles. Several previous manuscripts have highlighted the role of ABA as a negative regulator of adaptive responses of plants to soil flooding (Benschop et al., 2005; Shimamura et al., 2014). The data presented in this manuscript expand this knowledge and provide evidence that limited capacity for ABA biosynthesis correlates with enhanced RBOHD expression in hypoxic tissues and alters the ability to synthesize metabolites involved in anaplerotic pathways, subsequently contributing to an improved ability for survival under oxygen-depleted conditions. Moreover, greater transpiration, as observed in ABA-deficient genotypes, could contribute to improve water absorption under long-term waterlogged conditions and maintain leaf RWC, which has been identified as a physiological tolerance marker in citrus (Arbona et al., 2009; Rodríguez-Gamir et al., 2011).

## Data Availability Statement

The RNA-sequencing data generated in this study has been deposited into BioProject (accession: PRJNA684891).

## Author Contributions

CD, ZP, and VA conceived and performed the greenhouse experiments, collected the plant material, and analyzed the physiological and hormonal data. MG-G and VA performed the RNA extracts. JR and AG performed the polar metabolite analyses. VA and AG-C analyzed the semipolar metabolites. JM performed the bioinformatics RNA-seq analyses and GOEA. HC performed the gene promoter motif enrichment analyses. VA wrote the first draft of the manuscript. AG-C, CD, MG-G, and VA revised and edited the final version of the manuscript. All the authors read and approved the final version of the manuscript.

## Conflict of Interest

The authors declare that the research was conducted in the absence of any commercial or financial relationships that could be construed as a potential conflict of interest.

## Footnotes

1 https://transcriptomics.cloud

2 https://plants.ensembl.org/

3 https://www.dna.affrc.go.jp/PLACE/?action=newplace
