# Supplementary material for: Identification of ABA-Mediated Genetic and Metabolic Responses to Soil Flooding in Tomato (Solanum lycopersicum L. Mill)
Source: Front Plant Sci. 2021 Mar 5;12:613059. doi: 10.3389/fpls.2021.613059 (PMC7973378; doi:10.3389/fpls.2021.613059)
Supplement: Supplementary file 6 [file Data_Sheet_1.PDF]

Supplementary Material

Supplementary Figure S1. Tomato (*Solanum lycopersicum* L. Mill) cv. Lukullus (left) and cv. Ailsa Craig (right) along with their respective isogenic ABA-deficient lines *notabilis* and *flacca* (a). Plants used for the experiments and experimental induction of soil waterlogging (b).

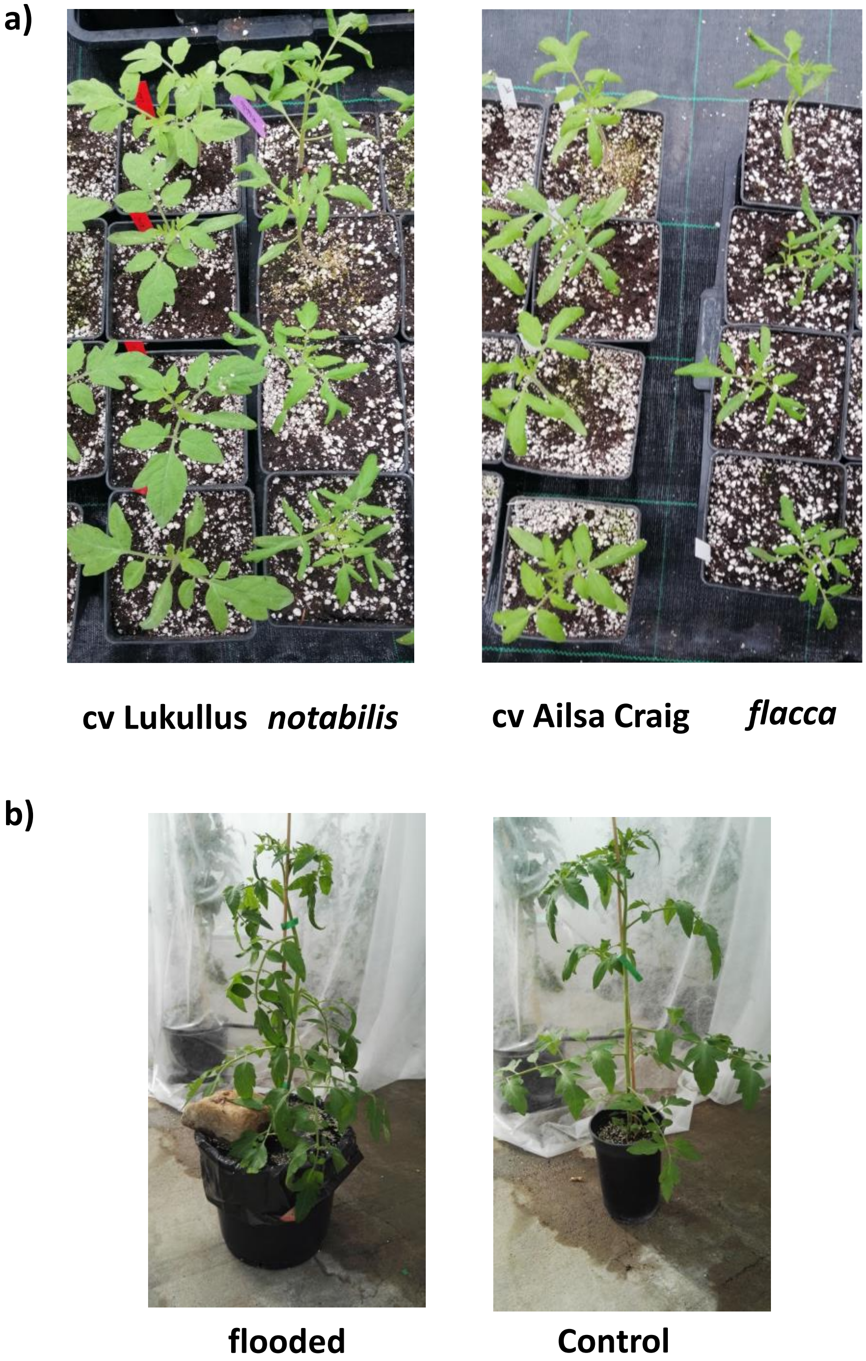

Supplementary Figure S2. Hormone concentration in shoots (upper pannel) and roots (lower pannel) in tomato cv. Lukullus and its isogenic ABA-deficient line *notabilis* (N=4). Statistical significance was assessed after two-tailed Student's t-test between control and flooded samples: significant at * 0.05, ** 0.01, *** 0.001, ns not significant.

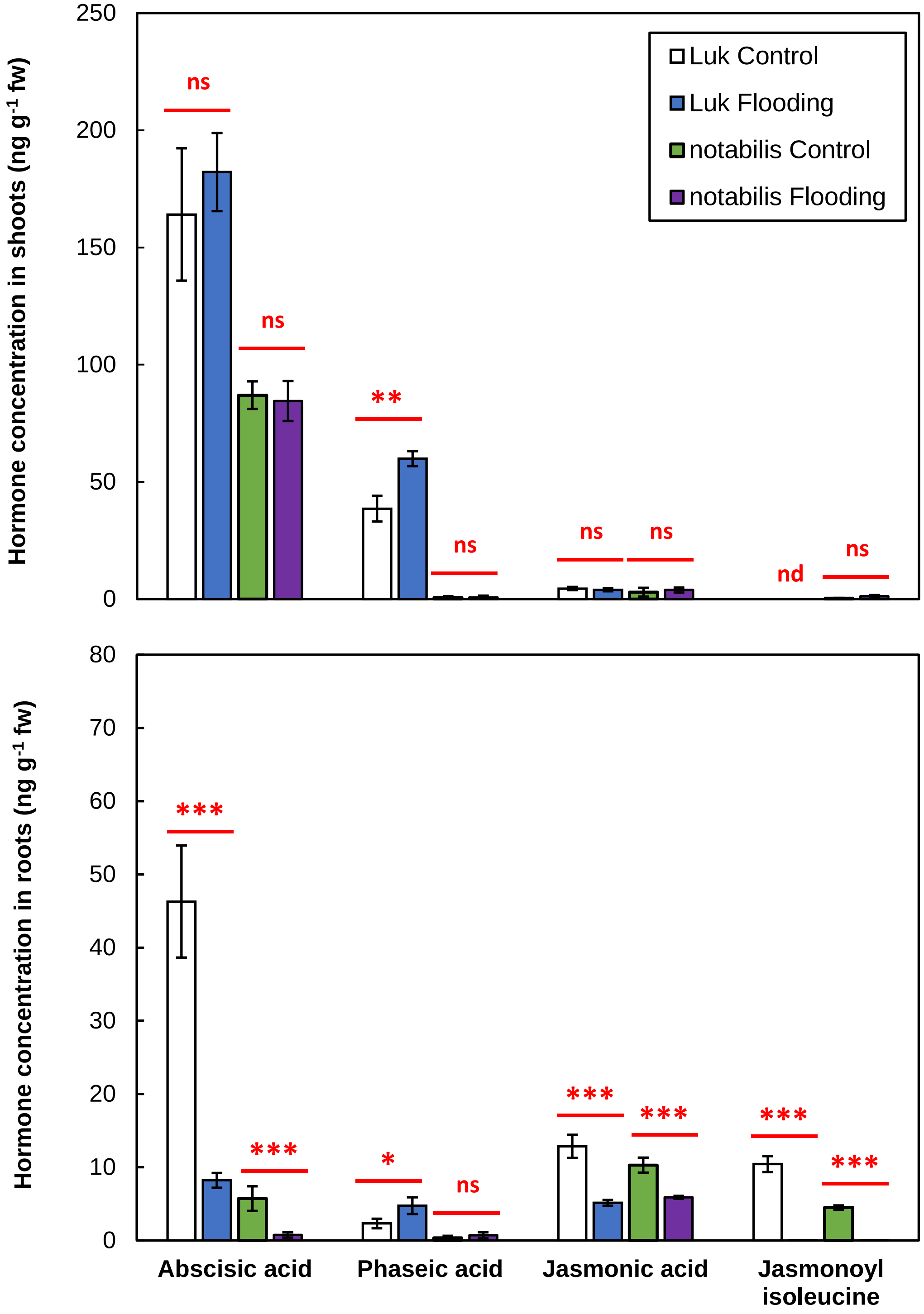

Supplementary Figure S3. Accumulation of hypoxia-specific metabolites in shoots and roots of tomato cultivars Lukullus and Ailsa Craig and their respective isogenic ABA-deficient lines *notabilis* and *flacca* (N=4). Statistical significance was assessed after two-tailed Student's t-test between control and flooded samples: significant at * 0.05, ** 0.01, *** 0.001, ns not significant.

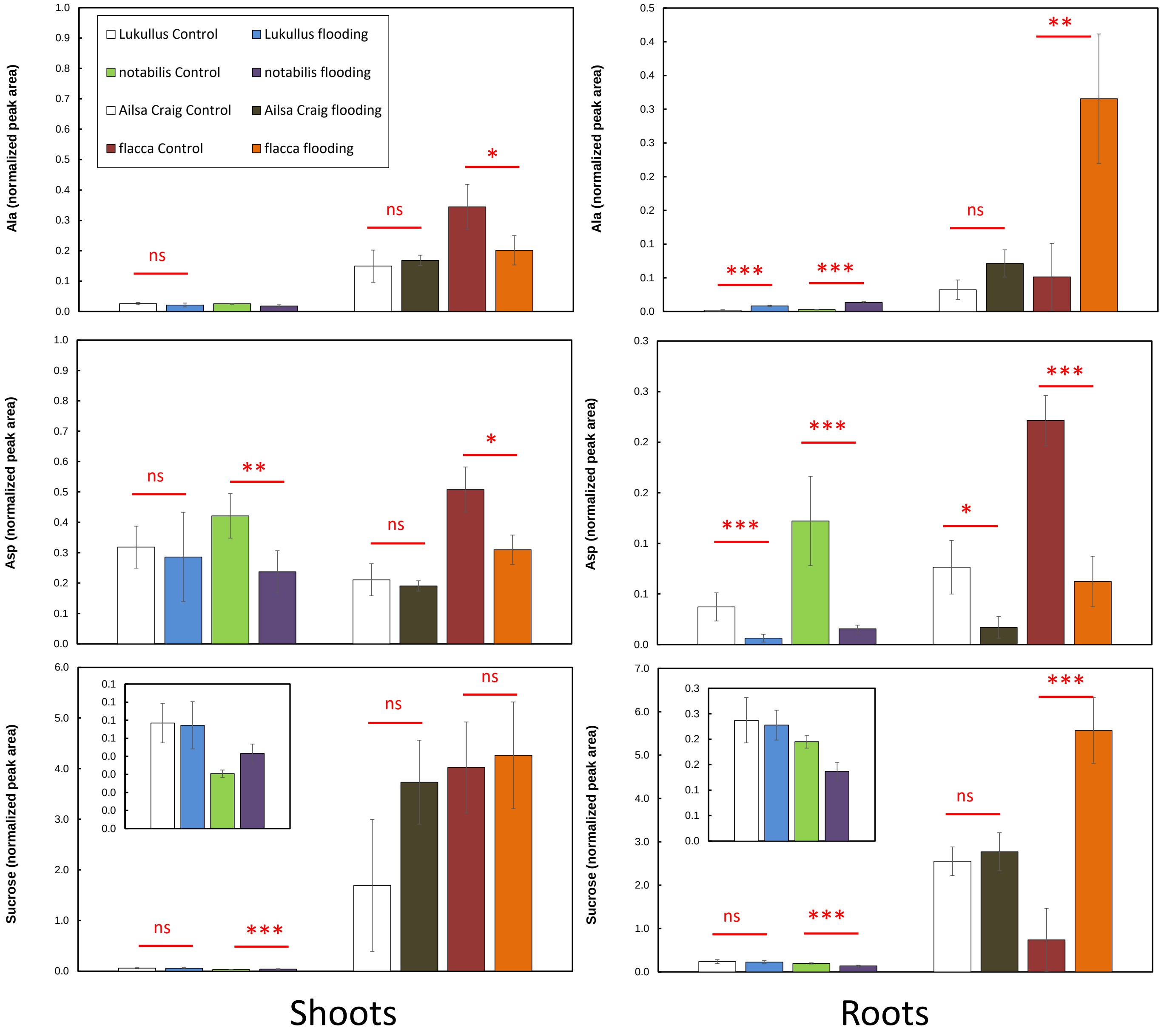

Supplementary Figure S4. Principal Component Analysis, scores plots of metabolite profiles in shoots and roots of control and flooded plants of cv. Lukullus (WT) and *notabilis* tomato plants (N=4).

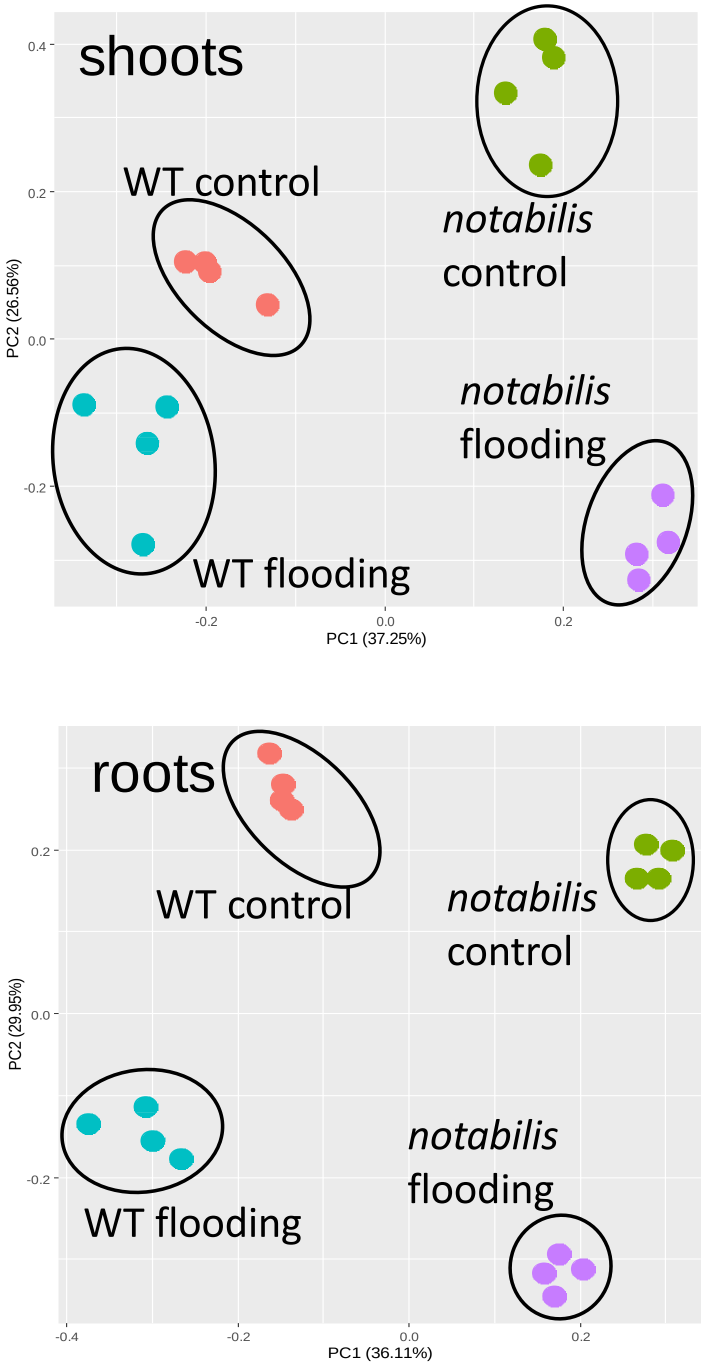

Supplementary Figure S5. JA-related transcripts. Each value is the mean value of three independent biological replicates.

	Roots WT	Roots *notabilis*	Shoots WT	Shoots *notabilis*	Roots WT/*notabilis*	Shoots WT/*notabilis*	
Solyc05g052620	1.11	1.01	0.20	nd	-0.10	nd	COI1
Solyc08g076930	-0.65	-1.03	-0.18	0.11	0.20	0.31	MYC2
Solyc08g005050	nd	-1.09	-0.36	0.19	nd	0.28	transcription factor MYC1
Solyc06g065820	nd	-2.81	nd	nd	nd	nd	ERF1
Solyc06g068930	2.44	2.10	nd	nd	-0.90	nd	Jasmonate ZIM domain protein g (AHRD V3.3 *** I3WTA5_NICAT)
Solyc03g122190	2.54	2.55	-0.56	0.52	0.43	0.15	Jasmonate zim-domain protein (AHRD V3.3 *** G7IIQ4_MEDTR)
Solyc12g009220	nd	-1.80	-1.97	nd	nd	nd	jasmonate ZIM-domain protein 1
Solyc01g005440	0.80	1.03	nd	0.18	-0.65	nd	Jasmonate ZIM-domain protein 3 (AHRD V3.3 *** B2XVS2_SOLLC)
Solyc01g103600	-1.36	-0.99	nd	nd	-0.08	nd	Jasmonate ZIM-domain protein 4 (AHRD V3.3 *** T1WMV9_TOBAC)
Solyc03g118540	2.01	2.20	nd	nd	-0.66	nd	Jasmonate ZIM-domain protein 7b (AHRD V3.3 *** T1WMB9_TOBAC)
Solyc12g049400	1.81	1.99	-0.99	nd	-1.76	nd	Jasmonate-zim-domain protein (AHRD V3.3 *** A0A167V6B0_CAMSI)
Solyc07g042170	3.54	2.46	-0.41	0.15	0.92	-0.44	Jasmonate-zim-domain protein (AHRD V3.3 *** A0A167V6B7_CAMSI)
Solyc05g007180	-2.49	-2.75	-0.57	nd	-0.16	nd	jasmonic acid 1
Solyc12g013620	-2.04	0.77	-0.16	-0.23	0.10	1.07	jasmonic acid 2
Solyc02g085730	-2.03	-2.09	nd	0.30	0.18	nd	allene oxide cyclase
Solyc04g079730	1.57	2.56	-2.60	nd	0.93	nd	allene oxide synthase
Solyc10g007960	-3.25	-3.94	nd	nd	-0.26	nd	Allene oxide synthase (AHRD V3.3 *** Q5NDE2_SOLTU)
Solyc11g069800	-0.39	-0.84	0.21	nd	0.08	nd	Allene oxide synthase (AHRD V3.3 *** Q8RW06_SOLTU)
Solyc11g011400	1.14	1.37	nd	nd	0.30	nd	Linoleate 9S-lipoxygenase (AHRD V3.3 --* LOXB_PHAVU)
Solyc10g086220	-0.51	-0.70	0.19	0.21	-0.74	-0.34	12-oxophytodienoate reductase
Solyc01g103390	1.42	0.50	0.82	0.46	-0.93	-1.73	12-oxophytodienoate reductase 2
Solyc07g007870	-1.40	-1.34	nd	0.15	-0.35	nd	12-oxophytodienoate reductase 3
Solyc11g032220	nd	nd	nd	nd	nd	nd	12-oxophytodienoate reductase-like protein (AHRD V3.3 *** G7K3S4_MEDTR)
Solyc11g032133	nd	0.87	nd	nd	nd	nd	12-oxophytodienoate reductase-like protein (AHRD V3.3 *** G7K3S4_MEDTR)
Solyc04g008470	-1.85	nd	nd	nd	nd	nd	Defensin (AHRD V3.3 *** C1K3M7_VIGUN)
Solyc07g017570	1.29	1.51	nd	nd	-0.21	nd	Defensin protein (AHRD V3.3 -** B1N682_SOLPI)
Solyc07g007750	-4.72	-3.64	-0.87	-1.14	-0.23	1.29	Defensin protein (AHRD V3.3 *** B1N678_SOLLC)
Solyc07g007760	-1.34	-0.45	-0.80	nd	-1.32	nd	defensin-like protein
Solyc10g009040	-3.67	-3.08	nd	nd	-1.10	nd	defensin-like protein (AHRD V3.3 --* AT4G21720.3)
Solyc07g009260	2.34	2.01	nd	-1.80	-3.27	nd	Defensin-like protein (AHRD V3.3 *-* A0A072TKV9_MEDTR)
Solyc07g009290	-0.77	nd	nd	nd	nd	nd	defensin-like protein (AHRD V3.3 -** AT1G19610.1)
Solyc07g009230	0.90	0.87	nd	-0.87	-1.95	nd	Defensin-like protein (AHRD V3.3 -** G7LB12_MEDTR)
Solyc07g009070	5.50	3.22	nd	nd	-0.80	nd	defensin-like protein (AHRD V3.3 *** AT1G19610.1)
Solyc07g009060	2.42	1.20	3.69	nd	-0.80	nd	defensin-like protein (AHRD V3.3 *** AT1G19610.1)
Solyc07g009040	3.15	1.53	nd	nd	-2.26	nd	defensin-like protein (AHRD V3.3 *** AT1G19610.1)
Solyc07g009030	2.90	0.77	nd	nd	-1.75	nd	defensin-like protein (AHRD V3.3 *** AT1G19610.1)
Solyc07g009020	4.59	0.67	nd	nd	-1.20	nd	defensin-like protein (AHRD V3.3 *** AT1G19610.1)
Solyc07g009090	2.37	1.39	nd	nd	-2.06	nd	defensin-like protein (AHRD V3.3 *** AT1G19610.1)
Solyc07g008980	2.29	1.11	nd	nd	-2.91	nd	defensin-like protein (AHRD V3.3 *** AT1G19610.1)
Solyc07g009100	1.72	0.71	nd	nd	-1.72	nd	defensin-like protein (AHRD V3.3 *** AT1G19610.1)
Solyc07g009050	2.45	0.93	nd	nd	-1.76	nd	Defensin-like protein, putative (AHRD V3.3 *** A0A061E6R0_THECC)

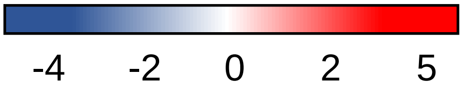

Log$_2$ (flooding/control) gene expression values
